# Reproductive health knowledge, attitudes and beliefs in young women with type 1 diabetes mellitus aged 15 – 25 years attending a tertiary centre multidisciplinary transition clinic: A descriptive study

**DOI:** 10.1186/1687-9856-2015-S1-P10

**Published:** 2015-04-28

**Authors:** Jesuina Noronha, Philip Bergman, Carolyn Allan, Susan Sawyer, Christine Muske, John Taffe, Emma Hurley, Adam Lamendola, Christine Rodda

**Affiliations:** 1 Department of Paediatric Endocrinology and Diabetes, Monash Children’s Hospital, Clayton, Victoria, Australia; 2 Department of Paediatrics, Monash University, Monash Children’s Hospital, Clayton, Victoria, Australia; 3 Department of Adolescent Medicine, Monash Children’s Hospital, Clayton, Victoria, Australia; 4 Departments of Endocrinology and Diabetes, Monash Health, Clayton, Victoria, Australia; 5 Monash Institute of Medical Research - Prince Henry's Institute, Clayton, Victoria, Australia; 6 Dept of Obstetrics and Gynaecology, Monash University, Clayton, Victoria, Australia; 7 Centre for Adolescent Health Royal Children’s Hospital, Parkville, Victoria, Australia; 8 The University of Melbourne; and Murdoch Children’s Research institute, Parkville, Victoria, Australia; 9 Centre for Developmental Psychiatry and Psychology, School of Psychology and Psychiatry, Monash University, Clayton, Victoria, Australia; 10 North West Academic Centre, University of Melbourne Sunshine Hospital, St Albans, Victoria, Australia

## 

To assess issues related to the existing reproductive health knowledge, attitudes and beliefs in 15 – 25 year old young women with type 1 diabetes mellitus (T1DM), we administered a questionnaire as sexually active adolescents with T1DM are at high risk of unplanned pregnancies and reproductive complications. Glycaemic control during adolescence is recognised as being poorer than during any other life stage. Periconceptional poor glycaemic control is associated with increased incidence of congenital malformations. This is a descriptive study in females aged 15 – 25 years attending our Young Adult Diabetes Service (YADS) clinic, a transition clinic at Monash Medical Centre, a conception to end of life tertiary health facility. Our study was undertaken between June 2011 and June 2013, when there were 173 eligible young women on our YADS clinic database. Data was collected on a cross-sectional basis from a web-based questionnaire on a sample of 100 female adolescents who provided consent to participate (58% of those eligible), using a modified reproductive health attitudes and behaviour (RHAB) questionnaire [1]. Almost half (48%) of respondents were sexually active, with a mean age of sexual debut of 16.9±1.8years (range 13 – 21yrs). Mean HbA1c was 8.9±1.6%. HbA1C in non participants was significantly higher (9.2±1.8%), although responders and non-responders were comparable on age and SES. Responses to questions related to their personal concerns about their future health risks, revealed that respondents perceived their risk and level of concern of unplanned pregnancies (18.7%) and STDs (8.7%), to be much lower than those of weight gain (59.3%) and blindness (57.1%). Despite high perceived benefits of prepregnancy counselling (PC) (78%), low PC delivery rates were reported by study participants (15%), although review of clinic check lists revealed documentation that 34% of study participants had received PC. Diabetic Nurse Educators (DNEs) were perceived to be the most useful source of health information (81% of respondents). We support recommendations of the National Health and Medical Research (NHMRC - 2011) that young adolescents need developmentally appropriate information from commencement of puberty, with a sensitive, proactive, preventative approach before these young women become sexually active, to enable them to make informed choices regarding reproductive health. However, engaging adolescent girls with T1DM to provide effective provision of such information remains challenging, even in an age appropriate specialised clinic setting.
